# Visual signs and symptoms in patients with the visual variant of Alzheimer disease

**DOI:** 10.1186/s12886-015-0060-9

**Published:** 2015-06-30

**Authors:** Pierre-François Kaeser, Joseph Ghika, François-Xavier Borruat

**Affiliations:** Department of Ophthalmology, University of Lausanne, Jules Gonin Eye Hospital, Avenue de France 15, CH-1004 Lausanne, Switzerland; Department of Neurology, University of Lausanne, CHUV, Lausanne, Switzerland

**Keywords:** Alzheimer, Reading difficulty, Homonymous hemianopsia, Simultanagnosia

## Abstract

**Background:**

Prominent visual symptoms can present in the visual variant of Alzheimer’s disease (VVAD). Ophthalmologists have a significant role to play in the early diagnosis of VVAD.

**Methods:**

We retrospectively reviewed the files of ten consecutive patients diagnosed with VVAD. All patients had a full neuro-ophthalmologic examination, a formal neurological and neuro-psychological testing, and cerebral MRI to confirm diagnosis. In addition, functional neuroimaging was obtained in seven patients.

**Results:**

The common primary symptom at presentation with all patients was difficulty with near vision (reading difficulty *n* = 8, “visual blur” in near vision *n* = 2), and difficulty writing (*n* = 3). Following assessment, impaired reading and writing skills were evident in 9/10 and 8/10 patients respectively. Median distance visual acuity was 20/25 and at near the median visual acuity was J6. Partial homonymous visual field defect was detected in 80 % (8/10) of the patients. Color vision was impaired in all patients when tested with Ishihara pseudoisochromatic plates, but simple color naming was normal in 8/9 tested patients. Simultanagnosia was present in 8/10 patients. Vision dysfunction corresponded with cerebral MRI findings where parieto-occipital cortical atrophy was observed in all patients. PET scan (5 patients) or SPECT (2 patients) revealed parieto-occipital dysfunction (hypometabolism or hypoperfusion) in all 7 tested patients

**Conclusions:**

Visual difficulties are prominent in VVAD. Dyslexia, incomplete homonymous hemianopia, preserved color identification with abnormal color vision on Ishihara, and simultanagnosia were all symptoms observed frequently in this patient series. Ophthalmologists should be aware of the possibility of neurodegenerative disorders such as VVAD in patients with unexplained visual complaints, in particular reading difficulties.

## Background

The number of patients presenting in clinic with reading difficulties increases with age. Most frequently, this complaint is due to either refraction abnormalities, corneal lesions, cataract, or a retinopathy. However, occasionally ocular examination shows no pathology with normal distance visual acuity; in such cases the origin of the reading difficulties is not evident. In 1988, Benson et al. reported a series of 5 patients with prominent visual complaints (reading difficulties) which had no evident ophthalmological origin. In this series all patients exhibited Balint’s syndrome (optic ataxia, simultanagnosia, oculomotor apraxia) and Gerstmann’s syndrome (acalculia, agraphia, finger agnosia, right-left confusion). Subsequently, posterior cortical atrophy was observed on MRI in 4/5 patients [1]. Posterior cortical atrophy (PCA) is a group of neurodegenerative disorders which affect primarily the parieto-occipital cortex [2]. Etiologies of PCA include Alzheimer’s disease (AD), corticobasal degeneration, dementia with Lewy bodies, subcortical gliosis, and prion diseases (Creutzfeldt-Jakob disease, fatal familial insomnia) [1–4]. The most frequent cause of PCA is AD, accounting for more than 80 % of cases. When AD presents with prominent visual symptoms, it is referred to as “Visual variant of Alzheimer’s disease” (VVAD) [5].

VVAD is a progressive neurodegenerative disorder in which visual symptoms are prominent, due to the predominance of parieto-occipital localization of pathologic changes. Neurofibrillary tangles and senile plaques similar to AD are present, and VVAD occurs usually in patients younger than those affected typically by AD [3, 4]. Because VVAD presents primarily with visual signs and symptoms, with a relative sparing of memory and other cognitive functions initially, VVAD patients present first to the ophthalmologist [1–3, 6–8].

Since the reported visual symptoms of VVAD are nonspecific, and routine ocular examination is usually non-conclusive, the ophthalmologist should be aware of the results of an array of simple clinical tests which are indicative of VVAD. This report outlines the ophthalmological results of a series of ten patients with major visual complaints which lead to the diagnosis of VVAD.

## Methods

We reviewed the charts of ten consecutive patients referred to the neuro-ophthalmology unit of the Jules-Gonin Eye Hospital between 2002 and 2008 for investigations of unclear visual loss, and ultimately diagnosed with VVAD.

All patients had a full neuro-ophthalmologic examination by one of us (FXB) including assessment of distance and near best corrected visual acuity, color vision (Ishihara pseudoisochromatic plates), oculomotility, visual field, slit lamp and fundus examination. Additional testing included simple mental calculations (additions, subtractions, divisions and multiplications of 1–2 digit numbers), writing abilities, reading one’s own handwriting, and assessment of simultanagnosia using the “Cookie Theft Picture” (Boston Diagnostic Aphasia Examination). Subsequently, all patients benefited from a formal neuropsychological testing and underwent complete neurological examination. Cerebral MRI was performed in all patients, and 7/10 patients underwent also functional cerebral imaging, either positron emission tomography (PET) or single photon emission computed tomography (SPECT).

This study was performed in accordance with the tenets of the Declaration of Helsinki and was approved by the Swiss Federal Department of Health (authorization # 035.0003-48). We confirm that consent to publish the clinical details of patients reported in this study was approved by the local ethics and research committee.

### Report of a case

**Fig. 1:**
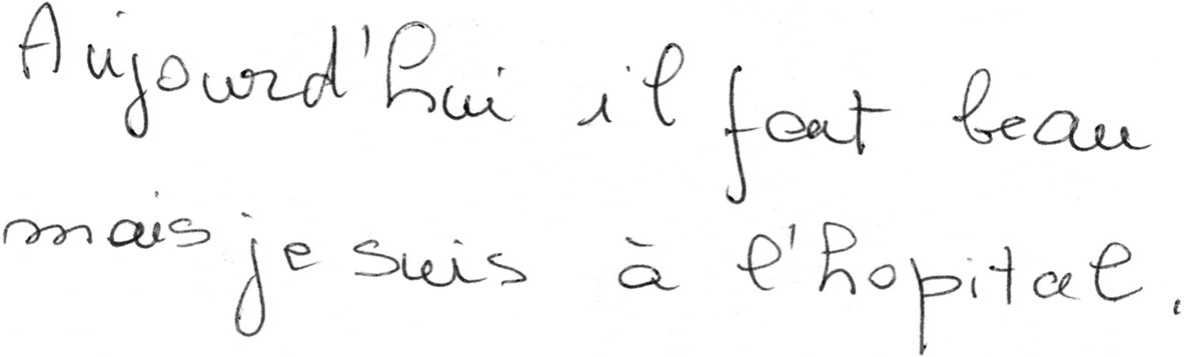
Patient 9. A 70 year-old woman complained of difficulty reading, but had a normal distance and near visual acuity. She wrote this sentence in French: “*Today the weather is nice, but I am in the hospital*”, but could not read her own handwriting 10 min later

**Fig. 2:**
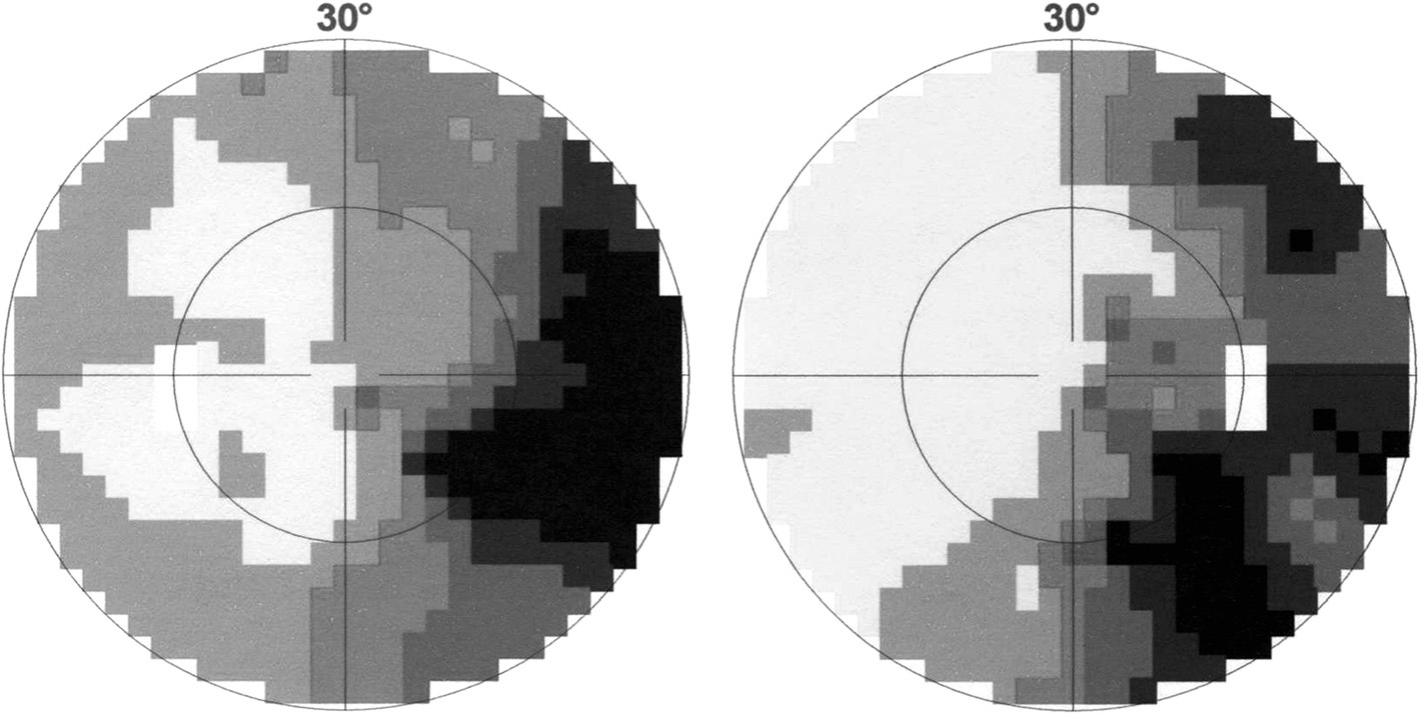
Patient 9. Result of computerized visual field examination revealed a right partial homonymous hemianopsia

**Fig. 3:**
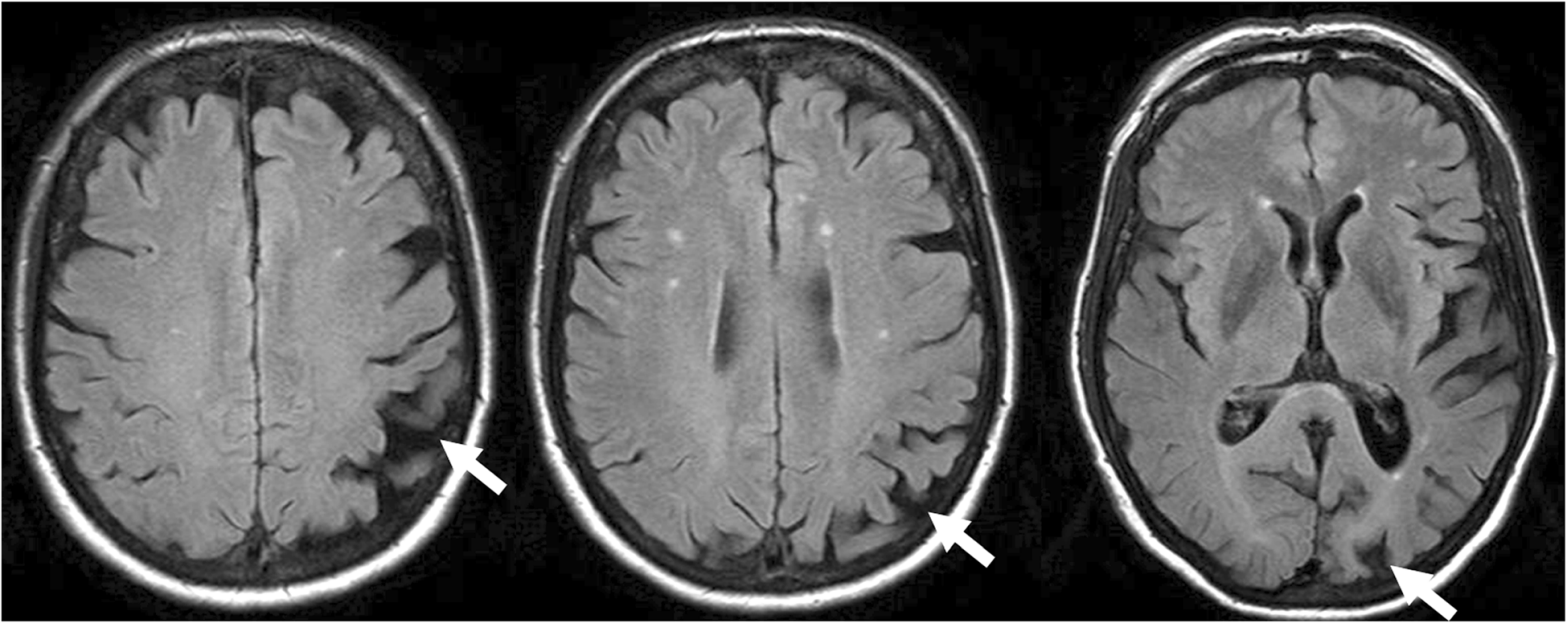
Patient 9. Cerebral MRI (T1) showed a left occipito-parietal atrophy (arrows)

**Fig. 4:**
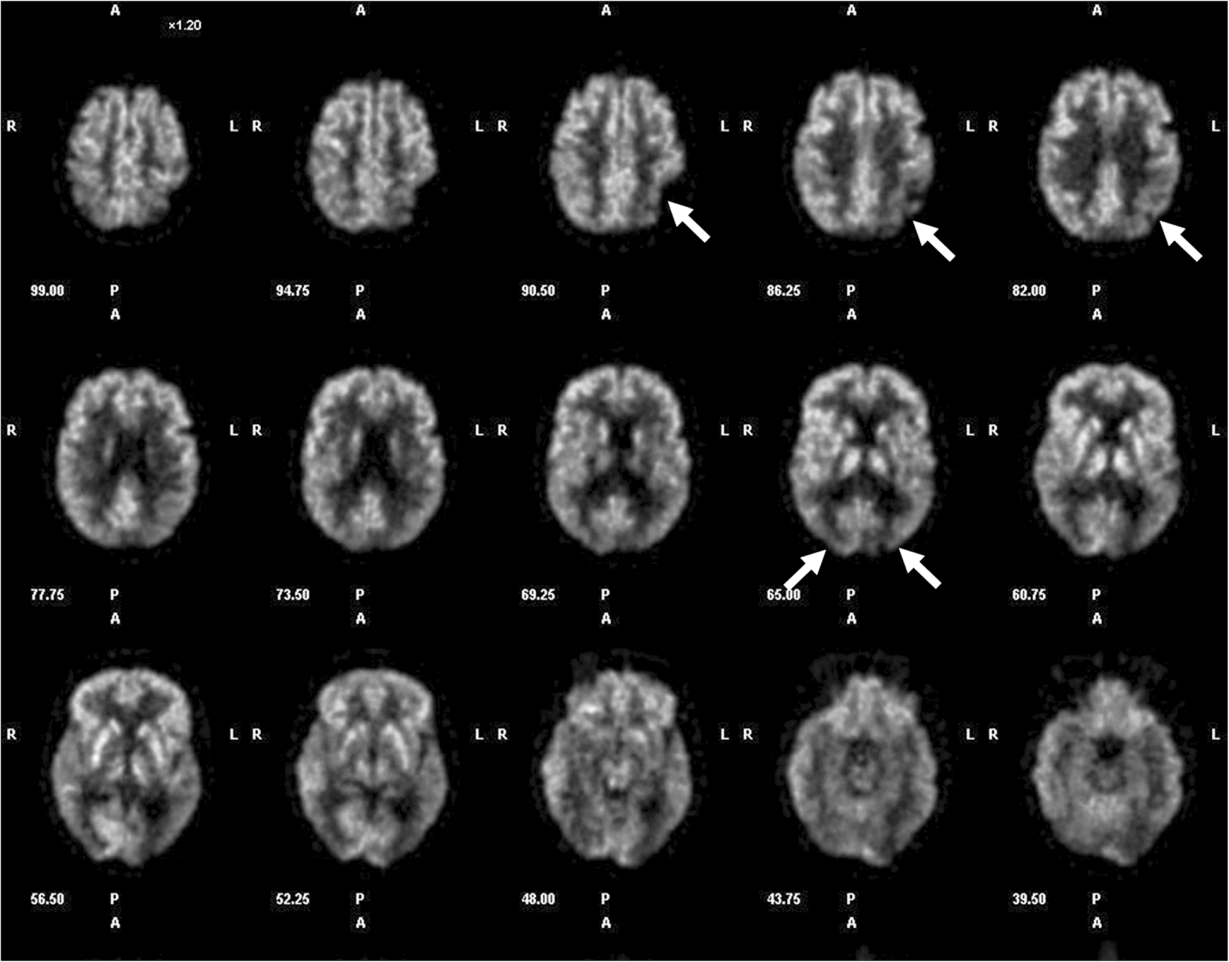
Patient 9. PET-scan disclosed a decrease in parieto-temporo-occipital metabolism (left > right) (arrows)

A 70 year-old woman (patient 9, Table 1) complained of difficulty reading for one year. Apart from a well-controlled systemic arterial hypertension, the patient was in good general health. She did not report any memory loss or orientation difficulty. Upon examination, best-corrected visual acuity (VA) was 20/25 at distance and J4 at near OU. Despite the relatively preserved visual acuity at near, she experienced major difficulties while reading. Biomicroscopy and fundus examination were normal. There was no dysgraphia (Fig. 1), but the patient could not read her own handwriting 10 min later. While the results of color vision testing were abnormal when tested with Ishihara pseudoisochromatic plates (0/13 correct answer), simple color identification and naming were normal and there was no dyschromatopsia on a 28-Hue test. There was no dyscalculia. Computerized visual field testing revealed a right partial homonymous hemianopsia (Fig. 2). Cerebral MRI revealed focal left parieto-occipital atrophy, with no other structural abnormality (tumor, stroke, demyelination) (Fig. 3). Results from both neurological and neuropsychological examinations were conclusive for VVAD. Results from a PET-scan showed decreased metabolism predominantly of the left parieto-temporo-occipital cortex (Fig. 4). Acetylcholinesterase inhibitor treatment was introduced with no favorable effect.

**Table 1:** Patients’ characteristics and radiological findings

Patient	Sex	Age (yrs)	Chief complaint (s)	Distance VA	Near VA	Visual field	Ishihara plates	Color naming	Dys-lexia	Dys-graphia	Simult-agnosia	Dys-calculia	Cerebral MRI (cortical atrophy)	Functional cerebral imaging (decreased metabolism/perfusion)
1	M	58	Difficulty reading & writing	20/25	J 16	L hemianopsia	0/13	normal	+	+	+	+	Posterior	N.a.
2	F	59	Difficulty reading	20/20	J 4	L hemianopsia	0/13	normal	+	+	+	+	Parieto-temporal	PET: R > L parietooccipital
3	F	61	Difficulty reading	20/20	J 12	R hemianopsia	0/13	normal	+	+	+	+	L > R posterior	N.a.
4	M	62	Difficulty reading	20/20	J 3	not reliable	0/13	abnormal	+	+	+	+	bilateral parietooccipital	SPECT: parietooccipital
5	M	62	Visual blur	20/50	J 3	R hemianopsia	6/13	normal	-	+	-	-	Posterior	PET: parietooccipital
6	F	65	Visual blur	20/63	J 8	L hemianopsia	3/13	N.a.	+	+	+	+	Posterior	PET: parietooccipital
7	F	68	Difficulty reading	20/25	J 6	R quadranopsia	0/13	normal	+	-	+	-	L > R partietooccipital	PET: parietooccipital
8	M	70	Difficulty reading & writing	20/63	J 8	R quadranopsia	0/13	normal	+	+	+	+	Posterior	SPECT: parietooccipital
9	F	70	Difficulty reading	20/25	J 4	R hemianopsia	0/13	normal	+	-	-	-	L partietooccipital	PET: L > R parietooccipital
10	F	82	Difficulty reading & writing	20/32	J 6	R caeco-central, L arcuate	0/13	normal	+	+	+	-	Posterior	N.a.

## Results

Ten patients were referred for evaluation of unexplained reading difficulties, ranging from “slow reading” to “impossible to read anything”. There were six women and four men. At the time of examination, the age of the patients ranged from 58 to 82 years (median 64 years). The onset of symptoms ranged from 5 months to 4 years before examination (median 18 months). General health was good in all patients without any pathology relevant to the loss of visual performances. The main visual complaint was impaired visual performances at near in all 10 patients. Difficulties in writing were also spontaneously reported in 3/10 patients. Only 3/10 patients spontaneously reported some memory loss, and a neurodegenerative disease was initially suspected in only one patient

At distance, the median visual acuity was 20/25 on Snellen chart (range 20/20–20/80), and at near the median visual acuity was J6 (range J1-J16). Results from Ishihara pseudoisochromatic plates testing were abnormal in all 10 patients (median 0/13, range 0/13–6/13), but simple color naming was normal in 8/9 tested patients. Formal visual field testing was performed in all patients under monocular conditions (computerized static visual field testing in 10 patients, additional Goldman kinetic manual perimetry in 4/10 patients). Results from visual field examination were reliable in only 6 patients, revealing incomplete homonymous hemianopia in 5/6 patients. Confrontation visual field testing showed partial homonymous defects in 3 of the 4 remaining patients. Slit lamp and fundus examination were normal in all patients.

Reading was impaired in 9/10 patients, and 8/10 had difficulty writing (Table 1). Simple mental calculation was impaired in 6/10 patients. Simultanagnosia (inability to interpret more than one element of a complex visual scene at the same time) was present in 8/10 patients, who failed to accurately describe the “Cookie Theft Picture” from the Boston Diagnostic Aphasia Examination. Optic ataxia (i.e. misreaching of objects under visual guidance) was detected in 9/10 patients. Results from formal neurological and neuropsychological examinations were abnormal in all 10 patients, revealing incomplete presentations of either Balint syndrome (optic ataxia, oculomotor apraxia, simultanagnosia) or Gerstmann’s syndrome (alexia, agraphia, acalculia, digital agnosia, left-right disorientation), partial prosopagnosia and various degree of memory deficits and apraxia. Results from the mini mental state examination (MMSE) were abnormal in 7/10 patients (median score 16/30, range 30/30–7/30; a score ≤ 24/30 being suspect of cognitive dysfunction). Cerebral MRI was performed in all patients, and revealed predominantly parieto-occipital cortical atrophy preserving the primary visual cortical areas in 10/10 patients (bilateral in 7/10 patients, unilateral left atrophy in 3/10 patients). No other structural abnormality (tumor, stroke, or demyelination) was detected. Results of PET (5 patients) and SPECT (2 patients) scans showed parieto-occipital dysfunction (hypometabolism or hypoperfusion) in all 7 tested patients.

The diagnosis of VVAD was made according to both clinical and radiological presentations. Specifically, no patient presented parkinsonism, complained of hallucinations, or exhibited extrapyramidal signs, myoclonus or ataxia. The clinical diagnosis of probable VVAD was made 5 months to 4 years after the onset of visual symptoms (average 18 months).

## Discussion

In patients aged 50 and above, reading disturbances are a frequent complaint, generally the origin of which is clear following a thorough ophthalmological exam. However, visual disturbances can also be a symptom of progressive dementia. Differentiating between dementia and another origin is based on clinical presentation, evolution, neuroradiological results, and sometimes results of brain biopsy. Alzheimer’s disease (AD) is the most common neurodegenerative disease, accounting for 50–65 % of all cases of dementia [9]. Visual impairment has previously been reported in AD, generally with advanced disease and severe cognitive impairment. Visual impairment in AD is understood to result from visual cortical involvement by neurofibrillary tangles and plaques, and include homonymous hemianopia, visual agnosia, Balint syndrome [7, 10]. Furthermore, progressive neural tissue loss in the retina (retinal ganglion cells) may contribute to the visual symptoms and visual loss in AD [11].

The visual variant of Alzheimer disease (VVAD) is a form of AD affecting patients of either sex in upper ranges of middle age. The early onset of visual symptoms and their prevalence in VVAD can be explained by the localization of the associated cortical atrophy, which in VVAD is predominately observed at the parieto-occipital lobes, whereas patients with typical AD show cortical lesions more prominently in the temporo-parietal lobes [9]. As opposed to the more frequent typical AD patients, cognitive impairment and/or memory disturbances are absent or mild, at least during the early stage of VVAD [1, 4–6]. Because visual symptoms are prominent manifestations of VVAD, ophthalmologists are often the first to evaluate these patients [1–3, 6–8]. However, the visual symptoms of VVAD at presentation are nonspecific, especially with respect to reading difficulties, “reading glasses do work anymore” or vague “visual blur”. In all our patients, the degree of visual impairment was marked, and did not correlate with the expected minor visual impairment or structural ocular abnormalities that are normally encountered in patients of this age. Such a discrepancy should raise the suspicion of VVAD [6, 8].

The signs and symptoms of VVAD depend on whether the dorsal (occipito-parietal) or ventral (occipito-temporal) visual processing pathway is the primary site of disease. The dorsal system is the most frequently involved (« where » pathway), resulting in Balint syndrome (simultanagnosia, oculomotor apraxia, optic ataxia), Gerstmann syndrome (agraphia, acalculia, right-left disorientation, finger agnosia), dressing apraxia, and aphasia. Lesions of the ventral system (« what » pathway) produce alexia without agraphia, visual object agnosia, and prosopagnosia (inability to recognize faces) [1–3, 6–8]. A complete Balint or Gerstmann syndrome is rare, and the most common feature for either syndrome is simultanagnosia and acalculia [1, 6]. In our series, 80 % of patients exhibited simultanagnosia and dyscalculia was observed in 6/10 patients.

As VVAD affects mostly the parieto-occipital cortex, involvement of the retrochiasmal part of visual pathway can occur. The occurrence of homonymous visual field defects in VVAD varies widely in the literature, with a prevalence ranging from 11 % to 78 % [3, 6, 8, 12–16]. Our results correspond well with the recent report by Pelak et al., in that homonymous visual field defects were present in 80 % of the patients reported here [17]. Computerized static visual field techniques are the most sensitive to detect small paracentral homonymous defects, but unfortunately require a very good cooperation of the patient, which is diminished in AD patients. Computerized visual field test was performed in all our 10 patients but was judged reliable and interpretable in only 6/10, disclosing homonymous defects in 5/6. In the remaining 4 patients (non reliable), additional testing with confrontational techniques revealed homonymous defects in 3/4. Visual neglect can co-exist with hemianopia. We did not specifically test our patients for hemineglect, but this can be easily assessed by the ophthalmologist using a letter cancellation or Bells test.

Abnormal results of color testing (Ishihara pseudo-isochromatic plates) were present in all our patients (median 0/13, range 0–6/13). Whereas these results could suggest moderate to severe dyschromatopsia, normal results of simple color naming were present in 8/9 tested patients. Such a discrepancy suggests an inability to derive form from color instead of altered color vision per se [6]. The abnormal results on Ishihara pseudoisochromatic plates test are then related to simultanagnosia instead of a true dyschromatopsia. Using a finger to trace the path on Ishihara color plates could help separating simultanagnosia from dyschromatopsia in VVAD patients. Despite obvious cortical atrophy in VVAD, dyschromatopsia is not typically found in these patients. Color identification is processed in the temporo-occipital lobe, area V4, which is not usually affected in VVAD [17].

When VVAD is suspected, cerebral imaging, formal neuropsychological and neurological evaluations are mandatory. Cerebral MRI or CT-scan is necessary to exclude any focal cerebral lesion (stroke, tumor, demyelination). In VVAD, neuroimaging techniques typically reveal occipital and/or parietal cortical atrophy, with a relative sparing of frontal and temporal cortex. This pattern was found in all our patients (Table 1). Functional cerebral imaging, by PET or SPECT, will usually reveal parietooccipital dysfunction, even in absence of anatomical cortical atrophy on CT or MRI [3, 6, 8, 12, 18]. Functional imaging was performed in 7/10 patients, and revealed occipital or occipitoparietal hypoperfusion (SPECT) or hypometabolism (PET) in all of them (Table 1). Neuropsychological evaluation was abnormal in all our patients and disclosed some signs of more global dementia in all our 10 patients, whereas only 3/10 patients spontaneously reported some memory impairment. Although a formal neuropsychological assessment is very useful, some higher cortical functions can be easily assessed by the ophthalmologist: difficulties to describe a complex scene (simultanagnosia) by showing a picture from a magazine, the « Cookie Theft Picture » (Boston Diagnostic Aphasia Examination) or Navon task stimulus; poor handwriting (dysgraphia); difficulties in reading despite adequate visual acuity (dyslexia); difficulties with some simple mental mathematical operations (dyscalculia); difficulties for the patient to reach under visual guidance an object with his hand (optic ataxia); and defective generation of saccades (oculomotor apraxia).

## Conclusions

Ophthalmologists should be aware of the possibility of VVAD in patients with unexplained visual complaints, in particular reading difficulties. Some simple clinical tests can strengthen a suspicion of neurodegenerative disorder, namely VVAD. The diagnosis needs to be confirmed by neurological, neuropsychological and neuroimaging examinations. Early diagnosis may carry a better prognosis facilitating earlier treatment. An early diagnosis also allows the organization of specialized social care and counselling for the patient and her/his family.
